# Metastatic Competency and Tumor Spheroid Formation Are Independent Cell States Governed by RB in Lung Adenocarcinoma

**DOI:** 10.1158/2767-9764.CRC-23-0172

**Published:** 2023-10-03

**Authors:** Nelson F. Freeburg, Nia Peterson, Dain A. Ruiz, Amy C. Gladstein, David M. Feldser

**Affiliations:** 1 Department of Cancer Biology, University of Pennsylvania, Philadelphia, Pennsylvania.; 2 Cell and Molecular Biology Graduate Group, University of Pennsylvania, Philadelphia, Pennsylvania.; 3 Abramson Family Cancer Research Institute, University of Pennsylvania, Philadelphia, Pennsylvania.; 4 Perelman School of Medicine, University of Pennsylvania, Philadelphia, Pennsylvania.

## Abstract

Inactivation of the retinoblastoma (RB) tumor suppressor in lung adenocarcinoma is associated with the rapid acquisition of metastatic ability and the loss of lung cell lineage commitment. We previously showed that restoration of RB in advanced lung adenocarcinomas in the mouse was correlated with a decreased frequency of lineage decommitted tumors and overt metastases. To identify a causal relationship for RB and its role in reprogramming lineage commitment and reducing metastatic competency in lung adenocarcinoma, we developed multiple tumor spheroid forming lines where RB restoration could be achieved after characterization of the degree of each spheroid's lineage commitment and metastatic ability. Surprisingly, we discovered that RB inactivation dramatically promoted tumor spheroid forming potential in tumors that arise in the *Kras^LSL-G12D/+^*; *p53^flox/^^flox^* lung adenocarcinoma model. However, RB reactivation had no effect on the maintenance of tumor spheroid lines once established. In addition, we show that RB-deficient tumor spheroid lines are not uniformly metastatically competent but are equally likely to be nonmetastatic. Interestingly, unlike tumor spheroid maintenance, RB restoration could functionally revert metastatic tumor spheroids to a nonmetastatic cell state. Thus, strategies to reinstate RB pathway activity in lung cancer may reverse metastatic ability and have therapeutic potential. Finally, the acquisition of tumor spheroid forming potential reflects underlying cell state plasticity, which is often predictive of, or even conflated with metastatic ability. Our data support that each is a discrete cell state restricted by RB and question the suitability of tumor spheroid models for their predictive potential of advanced metastatic tumor cell states.

**Significance:**

Members of the RB pathway are frequently mutated in lung adenocarcinoma. We show that RB regulates cell state plasticity, tumor spheroid formation, and metastatic competency. Our data indicate that these are independent states where spheroid formation is distinct from metastatic competency. Thus, we caution against conflating spheroid formation and other signs of cell state plasticity with advanced metastatic cell states. Nevertheless, our work supports clinical strategies to reactivate RB pathways.

## Introduction

Lung adenocarcinoma is the most common form of lung cancer, which causes approximately 1.7 million deaths per year (1). The *Kras^LSL-G12D/+^*;*Trp53^flox/^^flox^* (*KP*) mouse model is a gold-standard *in vivo* approach to study autochthonously arising cancers in a genetically controlled setting (2–5). Tumors that arise in the *KP* model recapitulate important features of human lung adenocarcinoma that include the myriad cell state changes associated with advancing malignancy (4). At a granular level, primary *KP* tumors can be categorized into those that have undergone cell state changes that promote malignant progression, marked by amplification of signaling downstream of KRAS^G12D^ through the MAPK pathway (6, 7) and a relatively rare subset of *KP* tumors that have progressed to become metastatically competent, which is identified by the lost expression of the lineage specifying transcription factor NKX2-1 and acquired expression of the nonhistone architectural transcription factor HMGA2 which is normally restricted to embryonic cell types (5, 8). Although the *KP* model captures these important barriers to tumor progression, the underlying molecular constraints are only beginning to be understood.

Members of the RB tumor suppressor pathway are frequently mutated in lung adenocarcinoma, but the effects of RB inactivation on tumor progression are not clear (9–11). The canonical role of the RB tumor suppressor is to constrain cell-cycle progression through the G_1_–S restriction point until receipt of appropriate mitogenic signaling cues (12, 13). However, an additional body of literature outlines noncanonical roles of RB that constrain cell state plasticity (14–19). To better understand the roles of RB in lung adenocarcinoma development, we developed the *Rb^XTR^* allele which allows reversible *Rb1* inactivation in the *KP* lung adenocarcinoma model (20). Inactivation of RB at early stages of tumor growth largely promoted cell proliferation and malignant progression in the absence of the typical requirement for aberrantly amplified MAPK signaling. This insight positioned RB as a major regulator of an early progression barrier to gaining malignant potential and supported canonical roles of RB in this disease. However, we additionally showed that RB inactivation promoted the loss of lung cell identity markers at early stages of tumor progression and accelerated the acquisition of poorly differentiated disease states and metastases. Thus, RB also plays critical noncanonical roles to regulate a late progression barrier that enforces lineage commitment and limits metastasis. In established lung adenocarcinomas that lacked RB expression throughout their development, RB reactivation was associated with high expression of markers of lung cell identity, downregulation of markers of metastatic competency, and a more differentiated cell state over the course of a 2-week period (21). The association of RB expression status and commitment toward lung cell states suggest that RB may directly regulate cell state commitment and metastatic progression. However, our inability to phenotype individual cancers while growing *in vivo* obscures a potential causal role for RB to reprogram cell state commitment and limit metastatic competency.

Tumor spheroid models are thought to preserve *in vivo* features of the primary tumors from which they are derived (22, 23). Here, cancer cells are isolated and suspended *ex vivo* in a semisolid matrix where they grow into hollow spherical structures that can be harvested and replated as continuously growing lines. Their ability to organize into spheres is reminiscent of organoid formation when culturing tissue stem cells in similar semisolid matrices (24). This has led many to postulate that tumor spheroid forming potential is a surrogate marker for the presence of stem cell–like character within tumor populations (25–27). To explore the causal relationship between RB, lineage plasticity, and metastasis in lung adenocarcinoma, we developed multiple tumor spheroid models from *KP;Rb^XTR^* tumors. We show that primary tumors from *KP;Rb^XTR^* mice have enhanced proclivity to establish long-term tumor spheroid cultures over primary tumors derived from *KP* mice. Once formed however, RB reactivation has no effect on the maintenance of tumor spheroid forming potential and only minimally limits the rate of tumor spheroid growth. Surprisingly, *KP;Rb^XTR^* tumor spheroids were equally likely to represent early-stage nonmetastatic primary tumors as they were to represent late-stage metastatic tumors. RB reactivation in metastatic tumor spheroids, despite having no effect on spheroid forming potential, dramatically reduced the ability of these cells to seed metastases when transplanted *in vivo*. These data indicate that although RB constrains both tumor spheroid forming potential and metastatic competency, these are independent and nonequivalent cell states.

## Materials and Methods

### Autochthonous Murine Lung Adenocarcinoma Studies

All animal use in this study was in compliance with the standards of the University of Pennsylvania Institutional Animal Care and Use Committee (#804774). *KP*;*Rb^+/+^* or *KP*;*Rb^TR^^/TR^* lung adenocarcinomas were initiated by forced inhalation of 60,000 plaque-forming unit of Cre-expressing lentivirus (PGK-Cre) in mice between 8 and 12 weeks of age. Mice were mixed B6J/129S4vJae (RRID: MGI:5474791). Viral particles were introduced intratracheally as described previously (4). Tumors were allowed to grow for 14 weeks before mice were sacrificed. Genetic reactivation of RB was accomplished by oral gavage of 200 µL tamoxifen at 20 mg/mL (Sigma, T5648) dissolved in a 90% corn oil/10% ethanol solution. Mice included in the study were sex balanced, and mice were randomly assigned to the treatment or control arm of the study. However, mice housed within a given cage were kept in the same treatment group to prevent unintentional tamoxifen exposure. Mice which were visibly overweight, underweight, or otherwise visibly abnormal were excluded from the study. Statistical power analysis was not used to determine the number of mice in the study. Instead, the number of mice included was based on an estimate of the number of tumors available per mouse and effect size produced by the treatment versus control. On the basis of this estimate, the minimal number of mice was used that would produce sufficient tumors to reveal statistically significant differences between treatment groups.

### Tumor Digestion

Individual tumors were picked from the lungs, chopped, and digested for 30 minutes in Hank's Balanced Salt Solution (HBSS) media containing final concentrations of 0.04% trypsin (Thermo Fisher Scientific, 25200056), 1.5 mg/mL collagenase IV (Worthington Biochemical, LS004188), and 7.65 U/mL Dispase (Corning, 354235). Tumor fragments were triturated with a p200 pipette tip and then strained through a 40 µm cell strainer.

### Tumor Spheroid Plating and Culture

A total of 12 µL Matrigel (Corning, 356234) was placed in a 24-well plate and incubated for 20 minutes at 37°C to solidify Matrigel. A total of 5,000 cells were resuspended in 6 µL of a 1:1 mixture of Matrigel and DMEM/F12 media (Gibco, 14170-112). The cells were then placed on top of the solidified Matrigel and incubated for an additional 20 minutes at 37°C to solidify. A final 12 µL Matrigel was placed on top and allowed to incubate 20 minutes longer at 37°C. The resulting Matrigel “plug” was then covered with 1 mL warm DMEM/F12 w/GlutaMAX (Gibco, 10565018) media.

### Tumor Spheroid Growth and Formation Assay

Tumor spheroids were plated at 5,000 cells per well as described above. To restore RB via the *Rb^XTR^* allele, media was treated with 4-hydroxy-tamoxifen (4OHT) or vehicle immediately upon plating. Growing spheroids were then imaged regularly using the Leica DMI6000 microscope. Tumor spheroid density was measured as the number of tumor spheroids in a representative 1 mm by 1 mm square within a microscope image. Measurement and spheroid counting was performed in ImageJ (NIH v 1.53t).

### Tumor Spheroid Single-cell Suspensions

Matrigel plugs containing tumor spheroids were broken up by trituration with Dispase (Corning, 354235) diluted 1:4 in media and incubated in their wells at 37°C for 30 minutes. Dispase was removed by centrifugation, and spheroid cells were incubated for an additional 5 minutes at 37°C in TrypLE (Gibco, 12604013). TrypLE was neutralized with FBS-supplemented media and removed by centrifugation. Cells were then resuspended in media and passed through a cell strainer. Cell counting was performed using a hemocytometer.

### Tumor Spheroid Bromodeoxyuridine Incorporation Assay

Tumor spheroids were plated at 5,000 cells per well and treated with 4OHT or vehicle as described above. Tumor spheroids were allowed to grow for 24 hours after plating before bromodeoxyuridine (BrdU) treatment. BrdU treatment and staining were performed using the APC BrdU incorporation Kit (BD 552598) as described in the product manual. BrdU treatment was performed for 3 hours at a final concentration of 10 µmol/L prior to collection, fixation, and permeabilization using the kit. After staining, BrdU incorporation was assessed using an Attune NxT flow cytometer (Thermo Fisher Scientific). Gating was performed to exclude doublets. Flow cytometry was analyzed using FlowJo (version 10.8.2, RRID: SCR_008520).

### Immunoblotting

Tumor spheroid cells were lysed in RIPA buffer, and lysate was resolved on a NuPage 4%–12% Bis-Tris gel (Invitrogen). Protein was transferred to a polyvinylidene difluoride membrane and blocked using LI-COR Intercept blocking buffer (Odyssey). Antibodies used were RB (Abcam 181616, RRID:AB_2848193) and β-actin (Cell Signaling Technology 4967S, RRID:AB_330288) diluted in PBS with 0.1% Tween-20 and 5% Milk. Secondary antibody incubation, chemiluminescence, and imaging were performed using the KwikQuant system (KindleBio). Conversion of raw images to black and white was performed using Adobe Photoshop (RRID: SCR_014199).

### *In Vitro* RB Restoration

Single-cell suspensions of primary tumor cells or tumor spheroid cells were plated in Matrigel as described above. RB was restored simultaneously with plating via treatment with 1 mmol/L 4OHT stock dissolved in ethanol for a final concentration of 500 nmol/L.

### *In Vivo* RB Restoration

RB was restored *in vivo* by oral gavage of tamoxifen. Two doses of 200 µL tamoxifen were administered 3 and 2 days prior to tumor harvest. Tamoxifen was dissolved at 20 ng/µL in a 90% corn oil, 10% ethanol solution.

### RNA Sequencing and Analysis of Tumor Spheroid Culture

Tumor spheroids were plated at 5,000 cells per well and allowed to grow for 10 days. A total of 72, 24, or 8 hours before harvest, tumor spheroids were treated with 4OHT to restore the *Rb^TR^* allele to its *Rb^R^* state. Tumor spheroids were harvested simultaneously, and RNA was extracted using the Qiagen RNeasy Micro Plus kit. RNA sequencing libraries were then prepared using the Illumina TruSeq stranded mRNA library prep kit and Illumina RNA Unique Dual Indices. Libraries were sequenced in single-end mode on a NextSeq 500 using a read length of 76 bp and index length of 8 bp, obtaining approximately 400 million reads, for 12.5 million reads per sample. Reads were pseudoaligned to the mm10 mouse genome (GRCm38) using the Kallisto pseudoaligner (version 0.46.2; ref. 28). Gene expression data were filtered to include only genes which had one or more count per million (CPM) across at least eight samples, and final gene expression was measured as Trimmed Mean of M component (TMM)-normalized CPM. Differentially expressed genes were identified using the Limma package in R (version 3.44.3; ref. 29). The significance threshold for differentially expressed genes was set at a fold-change cutoff of 4 and a Benjamini-Hochberg–adjusted *P*-value threshold of 0.05. Gene set enrichment analysis (GSEA) was performed using the GSEABase package in R (version 1.46.0).

### Metastatic Competency Group Signature and Human Patient Survival Analysis

To produce gene expression signatures representing each metastatic competency group, top differentially expressed genes were ranked by *P* value, and the 250 most significant genes defined a signature. Lung adenocarcinoma patient gene expression and survival data were obtained from the 2014 Nature dataset in The Cancer Genome Atlas (TCGA) via cBioPortal (11, 30). Single-sample GSEA (ssGSEA) was performed with each group signature against the patient gene expression data, and patients were rank ordered by enrichment for each signature. Next, the top and bottom 15% of patients enriched for each signature were extracted from the dataset, representing high and low enrichment groups. Survival of patients in high and low enrichment groups for each signature were plotted as Kaplan–Meier curves. The GSVA R package (version 1.46.0) was used to perform ssGSEA. Statistical analysis was carried out using the Survival (version 3.5) and survminer (version 0.4.9) R packages. Final visualization was performed using Prism software (GraphPad, Version 7.0c, RRID: SCR_002798).

### Tumor Spheroid Cell Intravenous Injection

Tumor spheroids were digested to a single-cell suspension as described above and resuspended in serum-free HBSS (Gibco, 14170-112) at a concentration of 50,000 cells per 200 µL media. A total of 200 µL of cells were injected into the tail veins of male mixed background mice (comparing Group 1 and Group 2 spheroids) or *FoxN1^Nu/Nu^* mice (comparing *Rb^TR^^/TR^* and *Rb^R^^/R^* Group 1 spheroids, Taconic, NCRNU-M, RRID:IMSR_TAC:NCRNU). All mice were age matched and were of equivalent weight. Mice were sacrificed for assessment of tumor burden 5 weeks after injection.

### Mouse Lung Sectioning, Imaging, and Tumor Quantification

Lungs were inflated with buffered 1:10 diluted formalin (Thermo Fisher Scientific 23-245685) via a needle inserted into the trachea. Lungs were then dissected from the mouse and immersed in buffered 1:10 diluted formalin overnight before transferring to 70% ethanol. Paraffin embedding, sectioning, and hematoxylin and eosin staining was performed by the Molecular Pathology and Imaging Core at the Perelman School of Medicine (Philadelphia, PA). Imaging was performed using a Leica DMI6000 microscope using a 20X and 5X objective. Tumors were counted while blinded to the treatment group of the mouse of origin.

### IHC

Paraffin-embedded lung sections were produced from mice intravenously injected with tumor spheroid single-cell suspensions as described above. Immunostaining was performed targeting Vimentin (Abcam, ab92547, 1:1,000, RRID: AB_10562134), Hmga2 (Cell Signaling Technology, 8179S, 1:500, RRID: AB_11178942), and Epcam (Abcam, ab71916, 1:150, RRID: AB_1603782) using Citrate-based antigen retrieval (Electron Microscopy Sciences, R-Buffer A, 62706-10). All targets were stained using ABC Reagent with biotinylated anti-rabbit secondary antibody (Vector Laboratories, PK-4001) and Immpact DAB (Vector Laboratories, SK-4105) according to product manual. Images were taken using a Leica DMI6000 microscope using a 20X and 5X objective.

### Statistical Analysis and Data Visualization

Other statistical analysis and data visualization were performed using the Prism software (GraphPad, Version 7.0c, RRID: SCR_002798) or the ggplot2 package (version 3.3.3, RRID: SCR_014601). in R (version 4.2.2)

### Data Availability

RNA sequencing data are available on Gene Expression Omnibus at accession number GSE240511. All other source data are available upon request.

## Results

### RB Inactivation Potentiates Lung Adenocarcinoma Spheroid Formation

**FIGURE 1:**
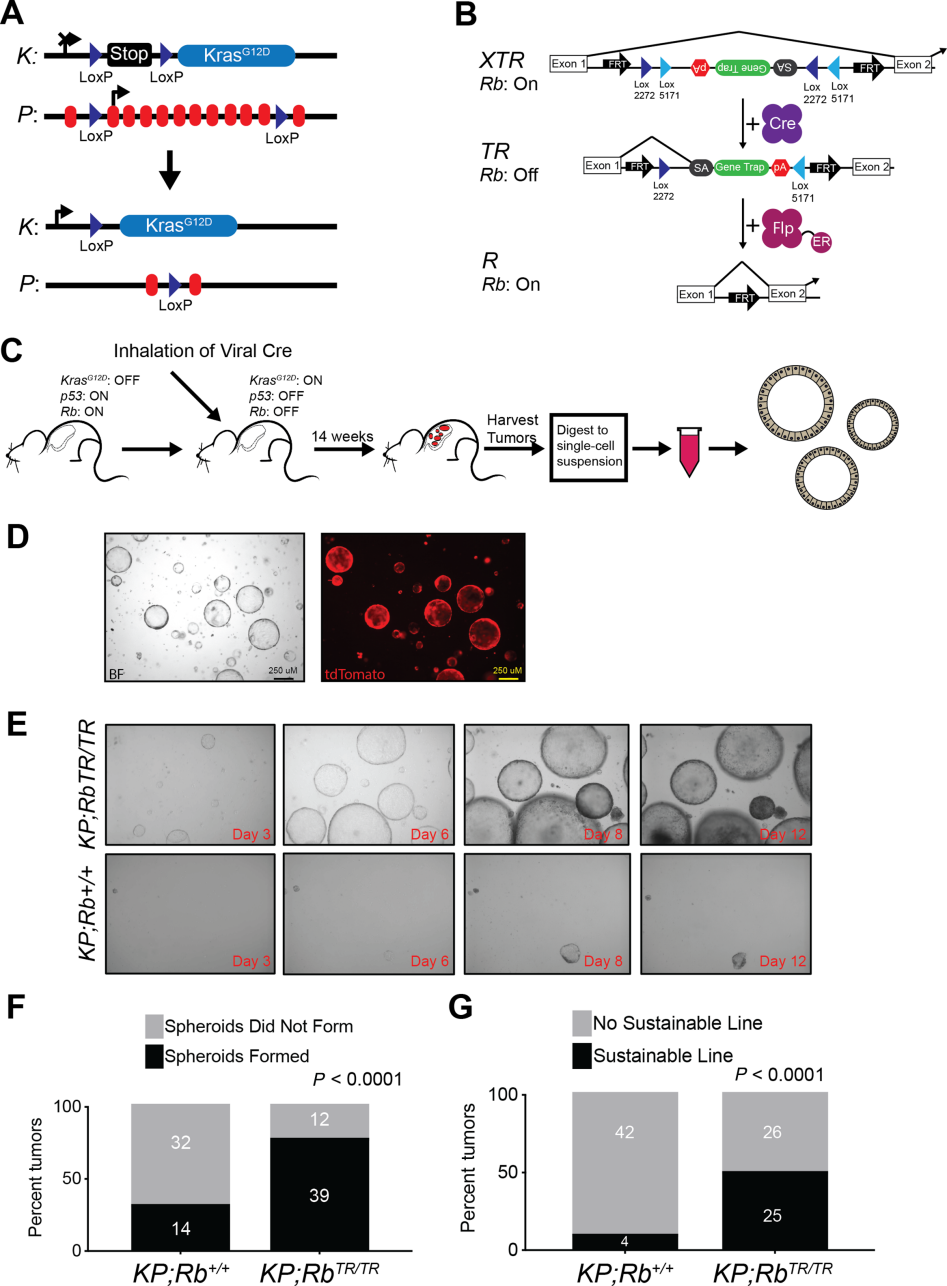
*Rb* inactivation is critical for tumor spheroid establishment. **A,** Schematic of *Kras^LSL-G12D^;Trp53^flox/^^flox^* autochthonous murine cancer model. Top: Stop cassette flanked by LoxP sites halts expression of hyperactive *Kras^G12D^*. *Trp53* is expressed and flanked by LoxP sites. Bottom: Upon endotracheal introduction of viral Cre recombinase, LoxP-flanked stop cassette is removed, enabling expression of *Kras^G12D^*. LoxP-flanked *Trp53* is also removed. **B,** Schematic of *Rb^XTR^* allele. Top: *Rb^XTR^* (expressed) allele. RB is expressed under control of its endogenous promoter. Between the first and second exons is an inverted gene trap consisting of a premature splice acceptor (SA), GFP, and poly-adenylation signal (pA). Middle, *Rb^TR^* (Trapped): Upon inhalation of viral Cre recombinase, recombination of two mutually incompatible LoxP sites results in stable inversion and activation of the gene trap, resulting in functional inactivation of the *Rb* gene. Bottom, *Rb^R^* (Restored): Constitutively expressed *Rosa26^FlpO-ERT2^* enables tamoxifen-dependent activation of estrogen receptor–conjugated Flp recombinase, resulting in deletion of the gene trap and restored expression of RB. **C,** Schematic of tumor spheroid production. Autochthonous tumor growth is induced in *KP* or *KP;Rb^XTR^* mice as described above. Tumors are harvested after 14 weeks and digested to produce a single-cell suspension. Tumor cell suspensions are plated in Matrigel, a soluble basement membrane matrix, allowing the formation of tumor spheroids. **D,** Tumor spheroid cells are marked by a cancer cell lineage marking *Rosa26^tdTomato^* allele. **E,***KP* or *KP;Rb^TR^^/TR^* tumor spheroid cells were plated in Matrigel and allowed to grow for up to 14 days. **F,** Contingency analysis of the number of tumor cell suspensions producing tumor spheroids, separated by *Rb* allele. Statistical significance was assessed by *χ*^2^ test. For *KP;Rb^+/+^* tumors *n* = 46 for, and for *KP;Rb^TR^^/TR^* tumors, *n* = 51. **G,** Contingency analysis of the number of tumor cell suspensions producing sustainable tumor spheroid lines, separated by *Rb* allele. Statistical significance was assessed by *χ*^2^ test. For *KP;Rb^+/+^* tumors *n* = 46 for, and for *KP;Rb^TR^^/TR^* tumors, *n* = 51.

To study the impact of RB inactivation during lung adenocarcinoma development and the subsequent reintroduction of the RB tumor suppressor in established cancer *in vivo*, we developed the *Rb^XTR^* allele and crossed it into the *KP* model creating *KP;Rb^XTR^* mice (Fig. 1A and B). Via endotracheal delivery, we transduced the lung epithelium with lentiviral vectors that express Cre recombinase. In the model, Cre induces expression of KRAS^G12D^, deletes p53, and inactivates *Rb1* gene expression by converting *Rb^XTR^* to the *Rb^TR^* allelic conformation. Reproducibly after 12–14 weeks, a subset of *KP;Rb^TR^* tumors progress to high-grade, poorly differentiated states that have metastatic potential (21). When crossed into the *KP;Rb^XTR^* model, the *Rosa26^FlpO-ER^* allele facilitates the conversion of the trapped *Rb^TR^* allele that is present in established tumors to the restored *Rb^R^* allele upon tamoxifen administration, thereby reactivating normal *Rb1* gene expression (31). Moreover, we incorporated a Frt-flanked *Rosa26^LSL-tdTomato^* allele to both mark tumor cell lineage and provide a visual indication of Flp activity due to tdTomato deletion (ref. 32; Supplementary Fig. S1). Restoration of RB expression via delivery of tamoxifen to *KP;Rb^XTR^* mice 12 weeks after tumor induction led to the progressive loss of high-grade, poorly differentiated primary tumors and reduced the number of observable distal metastases over the next 2-week period (21). Though this suggested that RB reexpression could rewire existing tumors toward a less advanced nonmetastatic cell state, establishing strict cause and effect relationships between RB and cell state changes was not possible due to the inability to phenotype existing tumors *in vivo* prior to RB reexpression.

To overcome this, we set out to develop tumor-derived spheroids from *KP;Rb^TR^* tumors that could be benchmarked against *KP;Rb^+/+^* tumors generated in an analogous manner (Fig. 1C and D). Individual tumors, which form clonally in the lung, were harvested and dissociated into single-cell suspensions. Cells were then embedded in Matrigel plugs and cultured in DMEM/F12 media supplemented with 10% FBS for up to 3 weeks. Cells from *KP;Rb^TR^^/TR^* tumors formed spheroids more readily than those from *KP;Rb^+/+^* tumors (Fig. 1E). Approximately 2 weeks following tumor cell plating in Matrigel, 76% of *KP;Rb^TR^^/TR^* tumors (39/51) showed evidence of tumor spheroid formation, while only 30% of *KP;Rb^+/+^* tumors (14/46) showed such growth (Fig. 1F). However, most *KP;Rb^+/+^* spheroids had poor spherical morphology, failed to thrive, and were unable to form new spheroids when dissociated and replated in new Matrigel plugs. In contrast, *KP;Rb^TR^^/TR^* tumor spheroids typically thrived in primary culture, had prominent spherical morphology, and could be sustainably serially passaged. Of all tumor cell suspensions plated, 49% *KP;Rb^TR^^/TR^* tumors formed sustainable tumor spheroid lines while only 8.7% of *KP;Rb^+/+^* did so (Fig. 1G). These unexpected results demonstrate that RB deficiency promotes tumor spheroid formation.

### RB Restoration Slows Tumor Spheroid Growth but does not Limit Tumor Spheroid Maintenance

**FIGURE 2:**
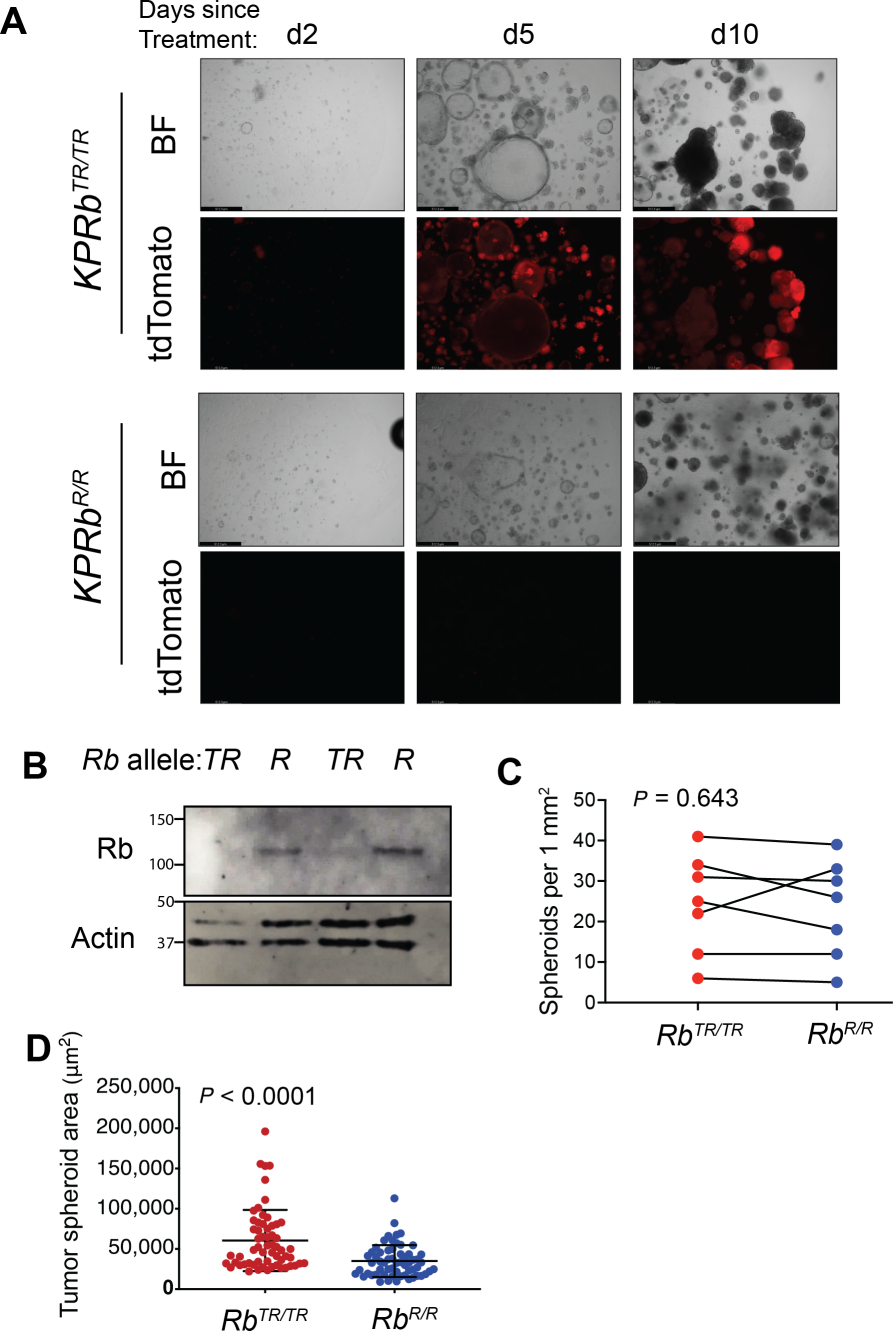
RB restoration limits tumor spheroid proliferation but not maintenance. **A,***KP;Rb^TR^^/TR^* tumor spheroid lines were plated in Matrigel and simultaneously treated with 4OHT (*KP;Rb^R^^/R^*) or vehicle (*KP;Rb^TR^^/TR^*). Spheroids were allowed to grow for 10 days. For *KP;Rb^TR^^/TR^* and *KP;Rb^R^^/R^*, the top row of images are brightfield, and to bottom row of images show tdTomato fluorescence. **B,** Western blot analysis for RB and β-ACTIN in two tumor spheroid lines treated with 4OHT (*KP;Rb^R^^/R^*) or vehicle (*KP;Rb^TR^^/TR^*). **C,** Quantification of tumor spheroid number in tumor spheroid lines treated with 4OHT (*KP;Rb^R^^/R^*) or vehicle (*KP;Rb^TR^^/TR^*). Significance was assessed by paired Student *t* test. For each treatment group, *n* = 7 tumor spheroid lines. **D,** Quantification of tumor spheroid size in tumor spheroid lines treated with 4OHT (*KP;Rb^R^^/R^*) or vehicle (*KP;Rb^TR^^/TR^*). Significance was assessed by Student *t* test. For each treatment group, *n* = 60 tumor spheroids from three tumor spheroid lines. Error bars represent mean ± SD.

That RB deficiency promotes tumor spheroid formation suggested that the status of RB could impact that ability of tumor cells to be maintained as tumor spheroids. To assess this, we capitalized on the reversibility of the *Rb^XTR^* allele to reactivate RB in established *KP;Rb^TR^^/TR^* tumor-derived spheroid lines. First, we plated tumor spheroid cells as a single-cell suspension in Matrigel and simultaneously restored RB by treatment with 4OHT. We then observed tumor spheroid growth over the next 7–10 days. Surprisingly, RB restoration did not diminish the ability of tumor spheroid cells to form new spheroids (Fig. 2A–C). However, *KP;Rb^R^^/R^* tumor spheroids grew slower and were less likely to be in S-phase than *KP;Rb^TR^^/TR^* spheroids indicating that the RB pathway *per se* remains intact (Fig. 2D; Supplementary Fig. S2). Therefore, while RB loss allows the acquisition of cell states which potentiate tumor spheroid formation, once the spheroid forming cell state is achieved, RB restoration is not sufficient to revert cells to a non–spheroid-forming state. Despite this, RB retains its canonical ability to regulate cell division, reestablishing tumor spheroid growth control after RB restoration.

### RB Restoration Prior to Spheroid Establishment Limits Spheroid Formation

**FIGURE 3:**
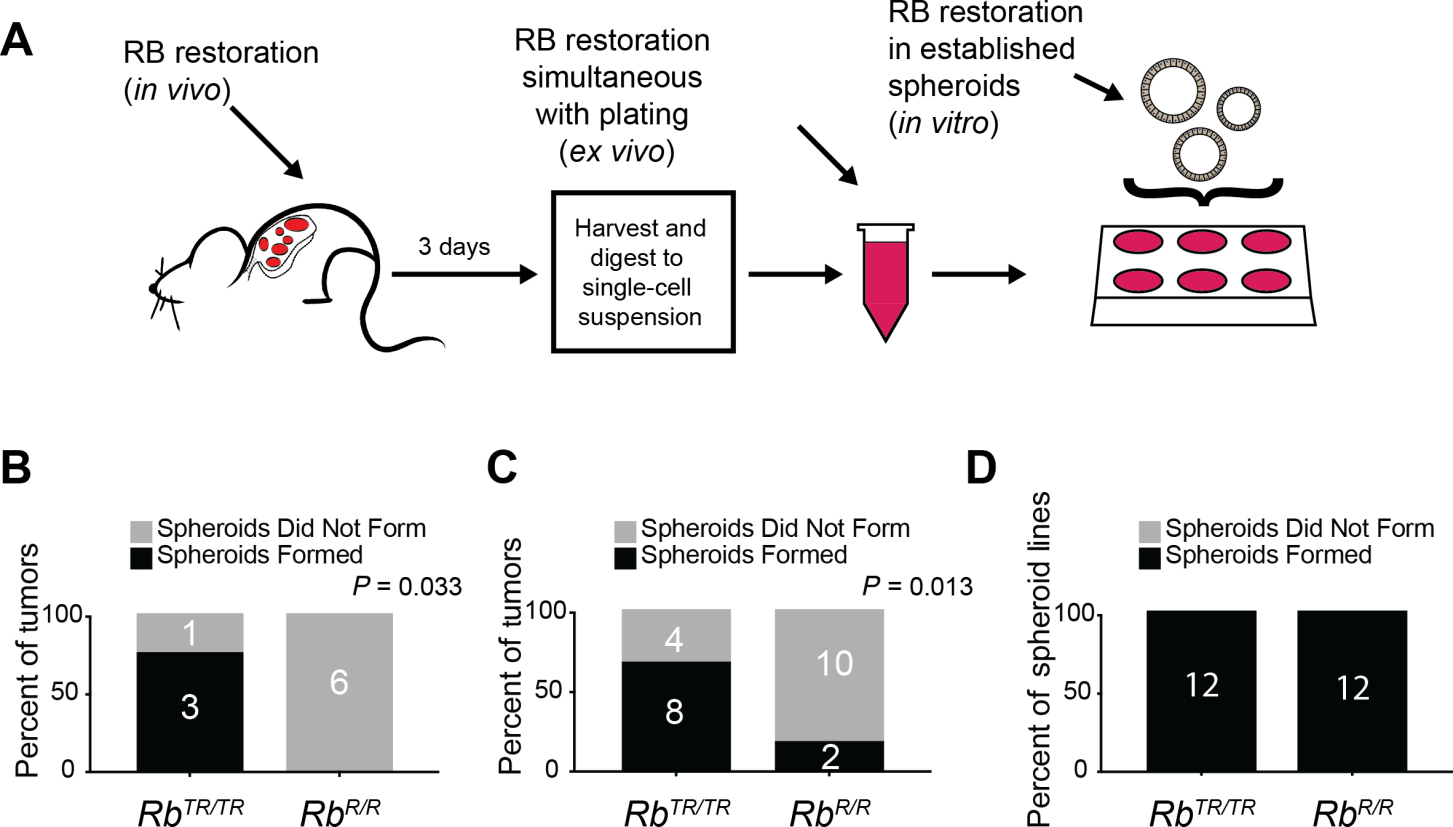
RB restoration prior to spheroid establishment limits spheroid formation. **A,** Schematic of RB restoration timepoints. RB can be restored *in vivo*, *ex vivo* prior to spheroid establishment in primary tumor cells, and simultaneous with plating in Matrigel, or *in vitro* in established tumor spheroid lines. **B,** Contingency analysis of the fraction of primary tumors from mice treated with tamoxifen (*Rb^R^^/R^*) or vehicle (*Rb^TR^^/TR^*) which formed tumor spheroids following plating of single-cell suspensions in Matrigel. Significance was assessed by *χ*^2^ test. For *KP;Rb^TR^^/TR^* tumors, *n* = 4 and for *KP;Rb^R^^/R^* tumors, *n* = 6. **C,** Contingency analysis of the fraction of primary tumor cell suspensions treated *ex vivo* with 4OHT (*Rb^R^^/R^*) or vehicle (*Rb^TR^^/TR^*) following plating in Matrigel. Statistical significance was assessed by *χ*^2^ test. For both groups, *n* = 12 tumors. **D,** Contingency analysis of the fraction of tumor spheroid lines treated *in vitro* with 4OHT (*Rb^R^^/R^*) or vehicle (*Rb^TR^^/TR^*) following plating in Matrigel. Statistical significance was assessed by *χ*^2^ test. For both groups, *n* = 12 tumor spheroid lines.

The complex effects of RB that strongly suppress tumor spheroid formation when cancer cells are cultured immediately *ex vivo* from primary tumors yet having no suppressive effect on tumor spheroid maintenance when reactivated in established tumor spheroid lines suggested that RB may act as a gatekeeper to tumor spheroid formation. To assess a gatekeeper role for RB in tumor spheroid formation, we reactivated RB either *ex vivo* immediately after harvest and simultaneous with plating, or *in vivo* 3 days prior to harvest. This approach was compared with RB reactivation in established *KP;Rb^TR^^/TR^* tumor spheroid lines as a benchmark (Fig. 3A). Comparing these three timepoints, restoration of RB *in vivo* reduced the rate of spheroid formation to the greatest extent, with 75% of control *KP;Rb^TR^^/TR^* tumors forming spheroids and 0% of *KP;Rb^R^^/R^* tumors forming spheroids (*P* = 0.033; Fig. 3B). RB restoration *ex vivo* in harvested tumors also diminished the rate of tumor spheroid formation, with 67% of *KP;Rb^TR^^/TR^* tumors and 17% of *KP;Rb^R^^/R^* tumors forming spheroids (*P* = 0.036; Fig. 3C). However, as seen before, RB restoration in established tumor spheroid lines *in vitro* had no effect on spheroid formation with 100% of both *KP;Rb^TR^^/TR^* and *KP;Rb^R^^/R^* tumors forming spheroids (Fig. 3D). These results suggest that RB inactivation enables cells to acquire a cell state that is amenable to tumor spheroid formation, but once tumor cells enter this spheroid forming state, RB restoration cannot revert the cells to a state that is incompatible with tumor spheroid maintenance.

### Tumor Spheroid Formation Represents a Distinct Cell State from Metastatic Competency

**FIGURE 4:**
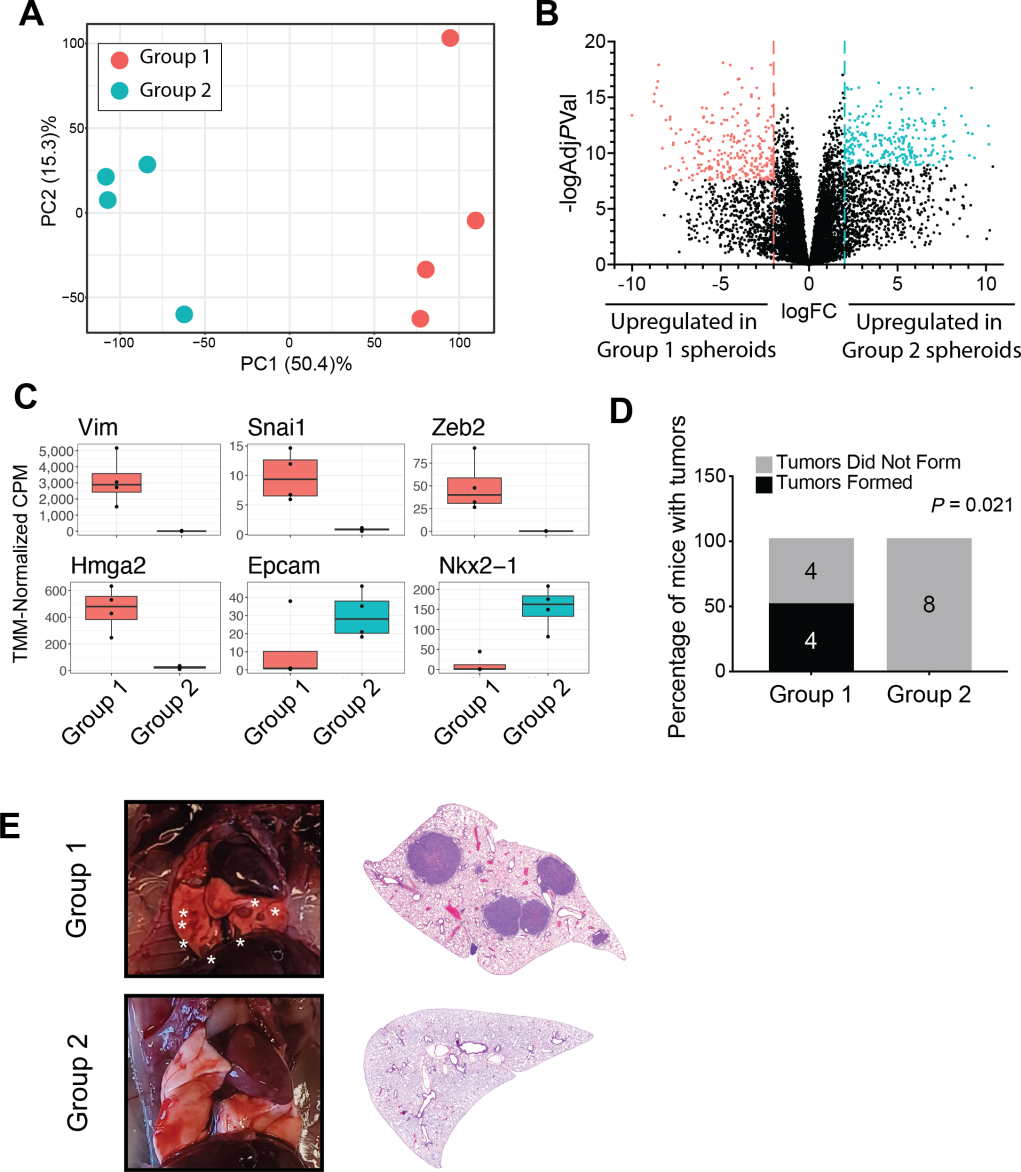
Spheroid formation represents a distinct cell state from metastatic competency. **A,** Principal component analysis plot for eight *KP;Rb^TR^^/TR^* tumor spheroid lines, colored by metastatic competency group membership. Red dots are members of Group 1, and blue dots are members of Group 2. Percentage values represent the percent of variation among samples represented by a given axis. **B,** Volcano plot of gene expression differences between metastatic competency groups. Each dot represents a gene, and colored dots are members of gene expression signatures representing each group. Red dots are members of the Group 1 gene expression signature, and blue dots are members of the Group 2 gene expression signature. **C,** Expression of key metastatic competency-related genes in each group. Gene expression is measured as TMM-normalized CPM. **D,** Contingency analysis of the number of mice injected with tumor spheroid single-cell suspensions which developed tumors after 6 weeks. Statistical significance was assessed by *χ*^2^ test. For both groups, *n* = 8 mice. **E,** Representative images and lung sections stained with hematoxylin and eosin from **D**. Lung section images were taken with a Leica DMI6000 using a 5X objective. White asterisks mark surface tumors.

Inactivation of RB in the *KP* model accelerates the acquisition of metastatic competency *in vivo* and tumor spheroid forming potential *in vitro*. To determine the extent to which these phenomena are linked, we performed RNA sequencing on eight *KP*;*Rb^TR^^/TR^* tumor spheroid lines selected on the basis of their robust growth as spheroids. Principal component analysis showed that tumor spheroid lines strongly separate along the first principal component, which accounts for 50.4% of the heterogeneity of the samples (Fig. 4A). Differential gene expression analysis between these two groups, that each had four tumor spheroid lines within, identified 3,285 differentially expressed genes (Fig. 4B). GSEA (MSigDB-Hallmark) comparing these two groups showed that they likely represent tumors that have (Group 1) or have not (Group 2) acquired metastatic competency. This result was not expected *a priori*, as all spheroid lines were derived from the same cancer model and genetic background. In fact, tumors from the same mouse did not necessarily fall into the same group, suggesting that metastatic group identification is almost entirely attributable to different evolutionary trajectories after tumor initiation. Group 1 tumor spheroids are highly enriched for programs associated the epithelial–mesenchymal transition, KRAS signaling, and Myc activity (Supplementary Fig. S3) and express high levels of the metastasis markers, such as Vimentin, TWIST1, and ZEB2 (Supplementary Fig. S4). In contrast, Group 2 spheroid lines lack mesenchymal marker gene expression and instead express EPCAM, the universal epithelial marker. Moreover, Group 1 spheroids express HMGA2, a functional marker of metastatic competency in lung adenocarcinoma. In contrast, Group 2 spheroids are highly enriched for the lung cell lineage markers NKX2-1 and SPC (Fig. 4C). These gene expression data suggest that *KP*;*Rb^TR^^/TR^* Group 1 spheroids represent metastatic primary tumors and *KP*;*Rb^TR^^/TR^* tumor Group 2 spheroids represent nonmetastatic primary tumors; a gene expression pattern reminiscent of tumor-derived cell lines from the *KP* model (5). Interestingly, gene expression signatures extracted from differentially expressed genes had variable ability to predict human lung adenocarcinoma patient survival. Signatures composed of the top 250 genes enriched in Group 1 or Group 2 were run through ssGSEA against the Nature 2014 lung adenocarcinoma TCGA dataset (11, 30). Comparing the survival of patients in the top and bottom 15th percentile for enrichment of each signature and found that the Group 2 signature significantly predicted patient survival (*P* = 0.021) whereas the Group 1 signature did not (*P* = 0.13; Supplementary Fig. S5A and S5B).

To test whether the gene expression differences we observed between tumor spheroid groups corresponded to actual differences in aspects of metastatic competency, we digested Group 1 and Group 2 tumor spheroids to single-cell suspensions and intravenously injected 50,000 cells into syngeneic mice. While an imperfect recapitulation of the entire metastatic cascade, tumor cell intravenous injection is a widely used assay for metastatic competency and accurately recapitulates major parts of the cascade including circulation, intravasation, and colonization of distal tissue (33–36). Dramatically, Group 2 spheroids were unable to form tumors at all, while 50% of mice (4/8) injected with Group 1 tumor spheroids seeded and expanded into multiple tumor masses in the lung and other distal locations (*P* = 0.021; Fig. 4D and E). These data support the gene expression patterns that predicted metastatic and nonmetastatic progression of the primary tumor from which these tumor spheroid lines were derived.

Interestingly, of the eight tumor spheroid lines sequenced, four were members of the metastatically competent Group 1 and four were in the nonmetastatic Group 2. That metastatically competent and nonmetastatically competent tumor cells were equally capable of forming tumor spheroids demonstrates that the capacity to form tumor spheroids is distinct from the ability to metastasize. Thus, tumor spheroid formation capacity cannot be conflated with metastatic ability.

### RB Restoration Reverses Metastatic Competency

**FIGURE 5:**
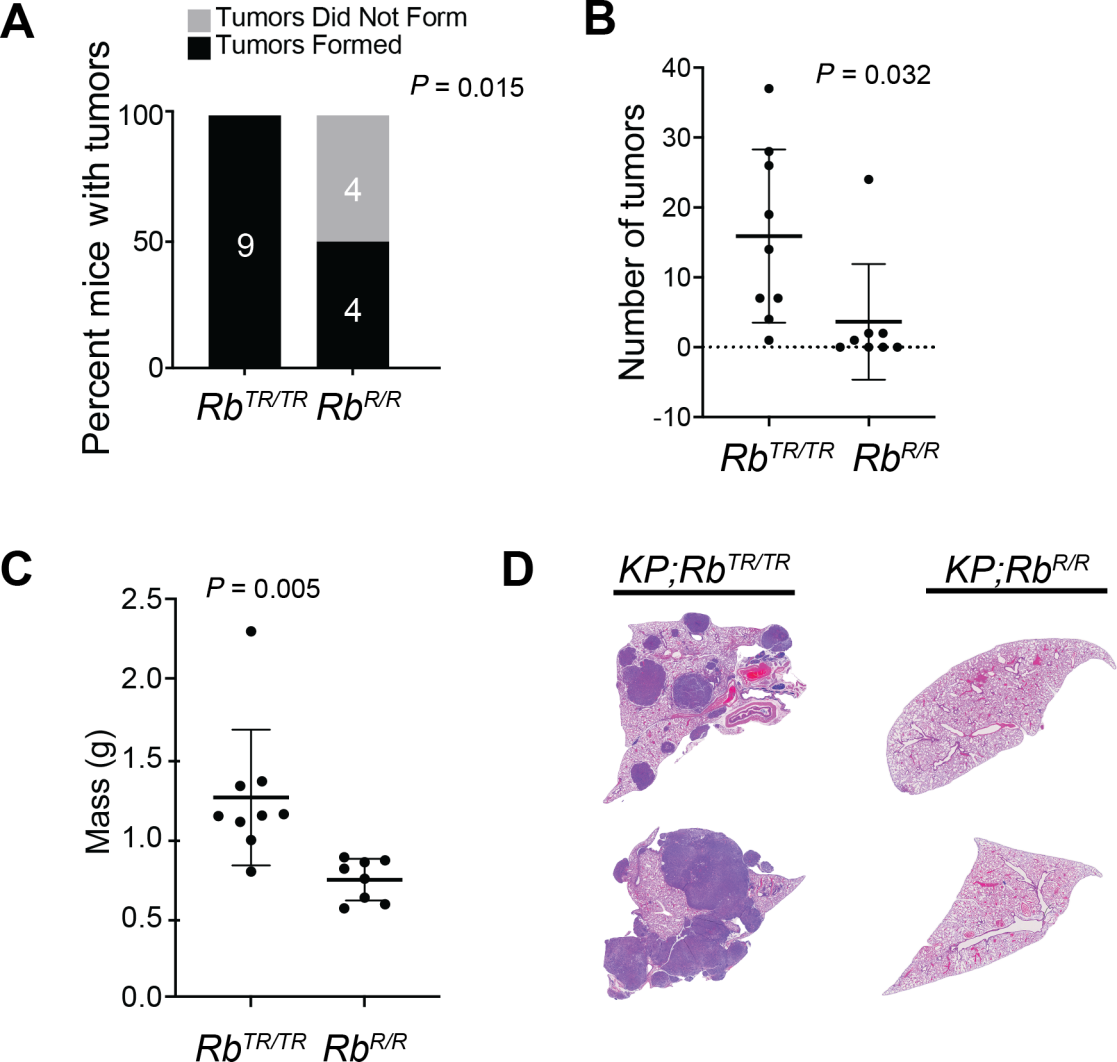
RB restoration reverses metastatic competency. **A,** Contingency analysis of the number of mice injected with single-cell suspensions produced from Group 1 tumor spheroids and treated with tamoxifen (*Rb^R^^/R^*) or vehicle (*Rb^TR^^/TR^*). Outcome of interest is tumor formation 5 weeks after injection. Statistical significance was assessed by *χ*^2^ test. For *KP;Rb^TR^^/TR^* mice, *n* = 9, and for *KP;Rb^R^^/R^* mice, *n* = 8. **B,** Number of tumors forming in mice five weeks after injection with single-cell suspensions produced from Group 1 tumor spheroids. Statistical significance assessed by Student *t* test. For *KP;Rb^TR^^/TR^* mice, *n* = 9, and for *KP;Rb^R^^/R^* mice, *n* = 8. Error bars represent mean ± SD. **C,** Lung mass in mice 5 weeks after injection with single-cell suspensions produced from Group 1 tumor spheroids. Statistical significance assessed by Student *t* test. For *KP;Rb^TR^^/TR^* mice, *n* = 9, and for *KP;Rb^R^^/R^* mice, *n* = 8. Error bars represent mean ± SD. **D,** Representative lung sections stained with hematoxylin and eosin from mice injected with single-cell suspensions produced from Group 1 tumor spheroids. Lung section images were taken with a Leica DMI6000 using a 5X objective.

Given the inability of RB restoration to affect tumor spheroid maintenance but its ability to limit tumor spheroid establishment, we questioned whether RB restoration could causally revert metastatic ability as was suggested in our previous work or whether RB was only acting as a gatekeeper to limit the development of metastatic competency. To test this, we intravenously injected 50,000 metastatically competent (Group 1) spheroid cells into the tail vein of recipient mice. After 3 days, we treated mice with either vehicle (corn oil) or tamoxifen via oral gavage. Five weeks after injection, mice were sacrificed and dissected to measure the abundance of tumor masses. All mice (9/9) that received Group 1 *KP*;*Rb^TR^^/TR^* tumor spheroid lines and were treated with vehicle control developed numerous metastatic nodules throughout the lung, pleural cavity, and/or distal sites. In contrast, mice that received Group 1 *KP*;*Rb^TR^^/TR^* tumor spheroid lines but were treated with tamoxifen to reactivate RB were significantly less likely to have tumor masses overall (*P* = 0.015; Fig. 5A), and those animals which did have tumors had far fewer and smaller masses than their control counterparts (Fig. 5B–D). These data strongly suggest that RB reactivation functionally reverts metastatic competency in *KP*;*Rb^TR^^/TR^* tumors and positions RB within both a gatekeeper and critical maintenance roles for metastatic cell states.

## Discussion

RB is a widely studied tumor suppressor with a well-understood role in constraining cell-cycle progression (12, 13, 37). In addition, RB has a well-documented but poorly defined ability to constrain cell state plasticity and the acquisition of metastatic competency, which often results from plasticity (14, 16–19, 38). Our results here are consistent with both of these roles of RB in lung adenocarcinoma. We showed that RB constrains both tumor spheroid formation and metastatic competency, likely via limiting cell state plasticity. When RB is lost in KP tumor cells, these cells develop an increased likelihood to form tumor spheroids and develop metastatic ability, indicative of heightened cell state plasticity. Prior to tumor spheroid establishment, RB restoration can limit the ability of KP tumor cells to form tumor spheroids. However, after RB-deficient spheroids are established, RB restoration merely slows cell proliferation. We speculate that RB prevents tumor spheroid formation and metastasis by maintaining a repressive epigenetic environment, which is alleviated when RB is lost. The de-repressed epigenetic environment allows cells to explore alternate cell states, including those permissive of metastasis or tumor spheroid formation. Importantly, that metastatically competent and metastatically noncompetent tumor cells are equally capable of forming tumor spheroids reveals that metastatic competency and tumor spheroid formation are distinct processes, even though they are both dependent on an RB-governed control of cell state plasticity. This conclusion is further supported by the observation that although RB restoration does not affect tumor spheroid maintenance, RB restoration in those same spheroid cells reduces their competency to form tumors upon intravenous injection.

It has been observed that a degree of cell state plasticity is needed for organoid/tumor spheroid formation (23–26). Cell state plasticity is also required for epithelial-derived tumor cells to gain migratory capabilities and leave the primary tumor site to begin the metastatic cascade (39–44). Because both processes are somewhat dependent on cell state plasticity, tumor spheroid formation is sometimes used as a readout for high-plasticity cell states which may give rise to metastases (23–26). However, our results show that while RB can constrain both the capacity for metastasis and tumor spheroid formation, these are distinct processes that are nonetheless fueled by the cell state plasticity afforded by RB inactivation. Thus, cell state plasticity, as measured by tumor spheroid formation capacity, is necessary but not sufficient for metastatic competency. We therefore suggest that caution should be taken in conflating tumor spheroid formation and other signs of cell state plasticity with metastatic competency. Even so, our results confirm the ability of RB to impede metastasis and support the use of clinical strategies to reactivate RB.

## Supplementary Material

Supplementary Data Figure 1 Frt-flanked and lineage-marking tdTomato allele

Supplementary Data Figure 2 RB restoration reduces BrdU incorporation.

Supplementary Data Figure 3 Hallmark gene sets enriched in Group 1 vs. Group 2.

Supplementary Data Figure 4 Tumors grown from Group 1 tumor spheroids express markers associated metastatic progression.

Supplementary Data Figure 5 Metastatic competency group signature enrichment and patient survival.
