# Supplementary material for: Metastatic Competency and Tumor Spheroid Formation Are Independent Cell States Governed by RB in Lung Adenocarcinoma
Source: Cancer Res Commun. 2023 Oct 3;3(10):1992–2002. doi: 10.1158/2767-9764.CRC-23-0172 (PMC10545537; doi:10.1158/2767-9764.CRC-23-0172)
Supplement: Supplementary Data Figure 1 — Frt-flanked and lineage-marking tdTomato allele [file crc-23-0172-s01.pdf]

Supplementary Data Fig. 1: Frt-flanked and lineage-marking tdTomato allele

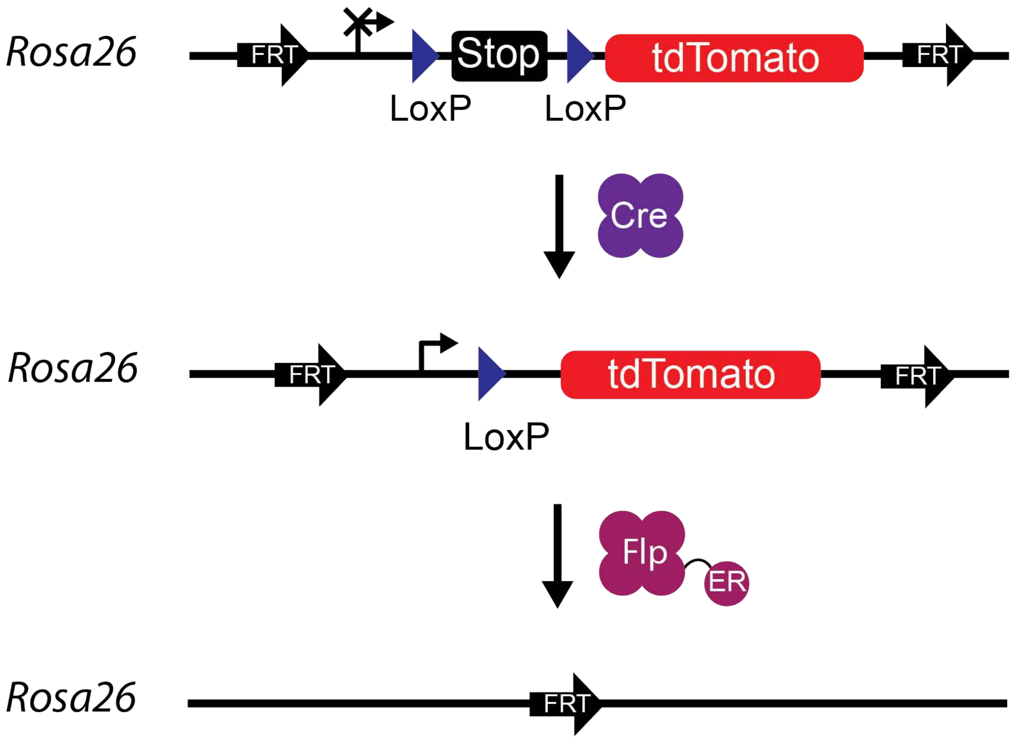

Schematic of Rosa26LSL-tdTomato allele with flanking Frt sites. A stop cassette flanked by LoxP sites is eliminated upon introduction of viral Cre, marking lung cancer cell lineage. Upon activation of Flp recombinase by tamoxifen, which also restores RB expression, recombination occurs between flanking Frt sites, eliminating the tdTomato and marking the reactivation of RB.
