# Supplementary material for: Metastatic Competency and Tumor Spheroid Formation Are Independent Cell States Governed by RB in Lung Adenocarcinoma
Source: Cancer Res Commun. 2023 Oct 3;3(10):1992–2002. doi: 10.1158/2767-9764.CRC-23-0172 (PMC10545537; doi:10.1158/2767-9764.CRC-23-0172)
Supplement: Supplementary Data Figure 2 — RB restoration reduces BrdU incorporation. [file crc-23-0172-s02.pdf]

Supplementary Data Fig. 2: RB restoration reduces BrdU incorporation

A

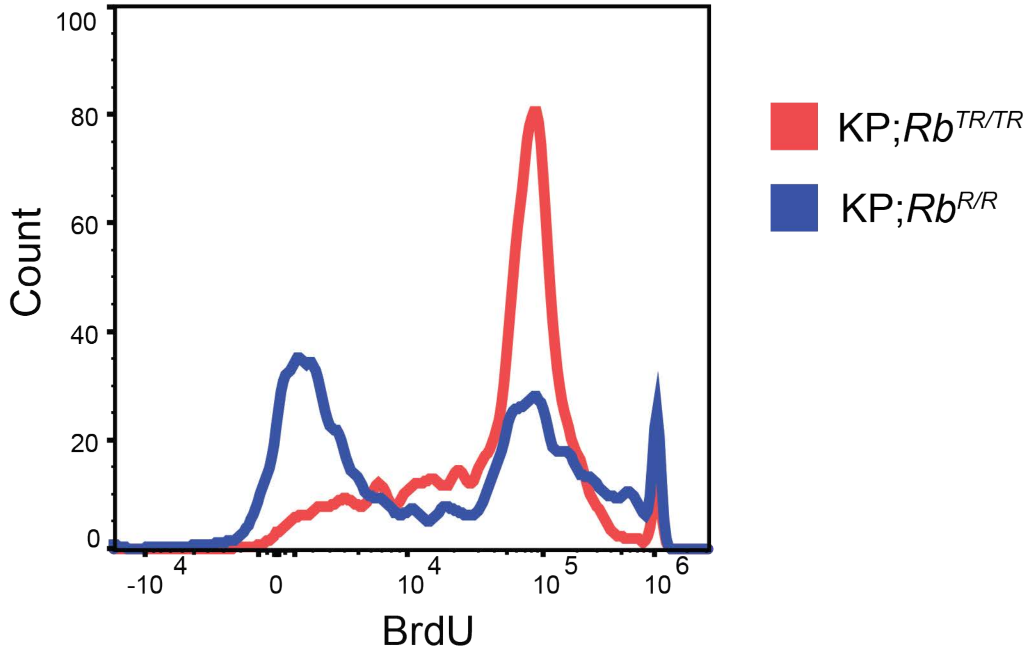

B

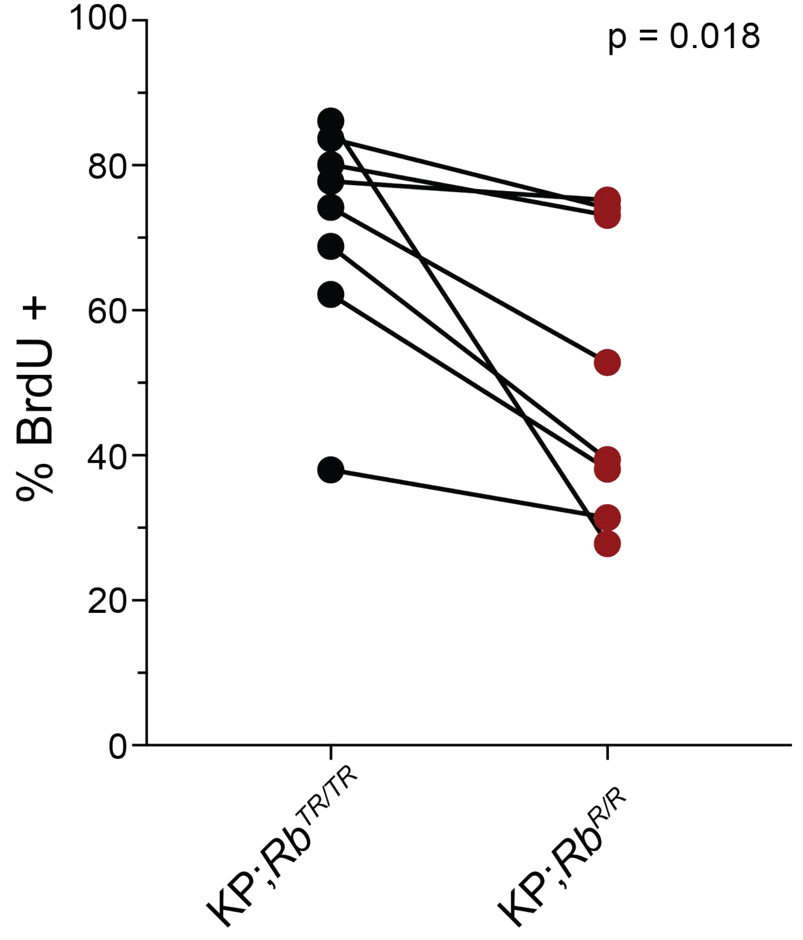

A. Representative flow cytometry histogram of BrdU fluorescence intensity in tumor spheroid lines in KP;RbTR/TR and KP;RbR/R tumor spheroids . RB was restored in tumor spheroids 72 hours prior to collection.

B. Quantification of fraction of cells which are BrdU-positive in 8 paired KP;RbTR/TR and KP;RbR/R tumor spheroid lines. BrdU positivity was assessed by flow cytometry, and significance was assessed by paired t-test.
