# Supplementary material for: Metastatic Competency and Tumor Spheroid Formation Are Independent Cell States Governed by RB in Lung Adenocarcinoma
Source: Cancer Res Commun. 2023 Oct 3;3(10):1992–2002. doi: 10.1158/2767-9764.CRC-23-0172 (PMC10545537; doi:10.1158/2767-9764.CRC-23-0172)
Supplement: Supplementary Data Figure 3 — Hallmark gene sets enriched in Group 1 vs. Group 2. [file crc-23-0172-s03.pdf]

Supplementary Data Fig. 3: Hallmark gene sets enriched in Group1 vs. Group 2

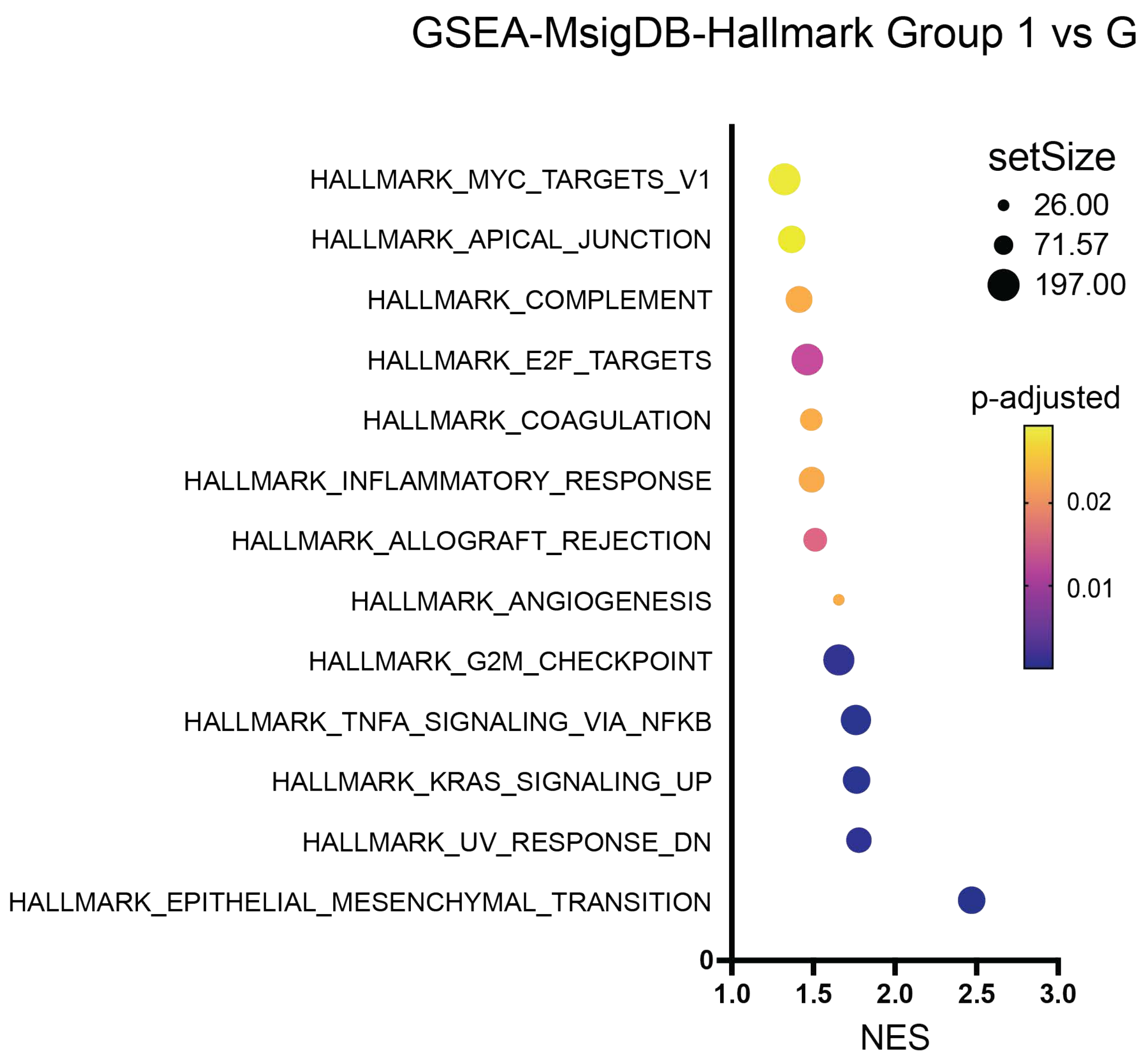

Top terms from MSigDB-Hallmark, ranked by normalized enrichment score (NES), which are enriched when performing gene set enrichment analysis (GSEA) comparing Group 1 to Group 2. Dot size indicates gene set size. Dot color indicates Benjamini-Hochberg adjusted p-value. GSEA was performed using the GSEAbase package in R.
