# Supplementary material for: Metastatic Competency and Tumor Spheroid Formation Are Independent Cell States Governed by RB in Lung Adenocarcinoma
Source: Cancer Res Commun. 2023 Oct 3;3(10):1992–2002. doi: 10.1158/2767-9764.CRC-23-0172 (PMC10545537; doi:10.1158/2767-9764.CRC-23-0172)
Supplement: Supplementary Data Figure 4 — Tumors grown from Group 1 tumor spheroids express markers associated metastatic progression. [file crc-23-0172-s04.pdf]

Supplementary Data Fig. 4: Tumors grown from Group 1 tumor spheroids express markers associated metastatic progression

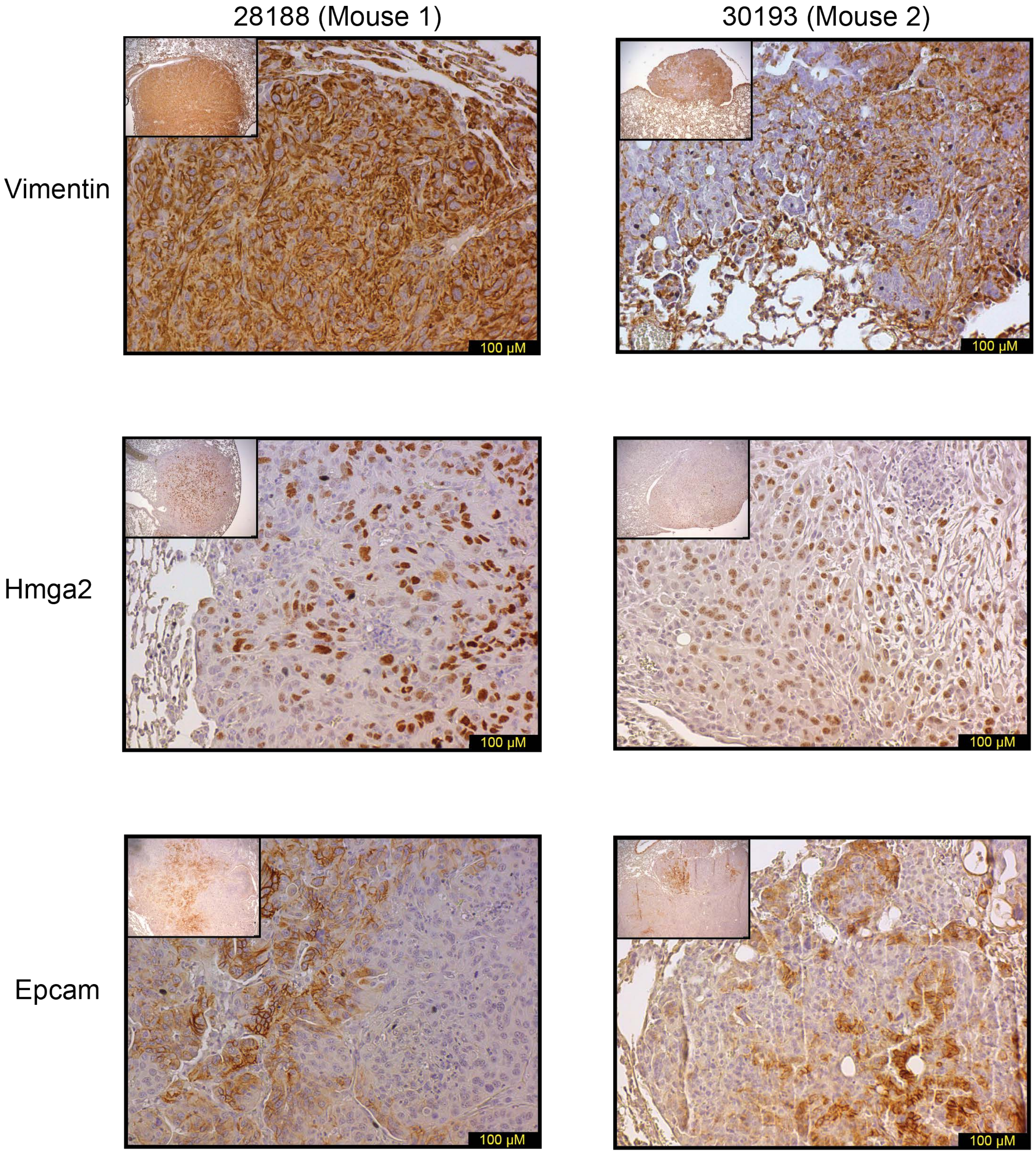

Vimentin, Hmga2, and Epcam immunohistochemistry on FoxN1Nu/Nu mouse lungs containing tumors seeded by intravenous injection of tumor spheroid single cell suspensions. Tumors were allowed to grow for 6 weeks following injection. Lungs were harvested, formalin-fixed, and paraffin-embedded. Main images were taken with a 20X objective, and insets were taken with a 5X objective. Scale bar is 100 M.
