# Supplementary material for: Metastatic Competency and Tumor Spheroid Formation Are Independent Cell States Governed by RB in Lung Adenocarcinoma
Source: Cancer Res Commun. 2023 Oct 3;3(10):1992–2002. doi: 10.1158/2767-9764.CRC-23-0172 (PMC10545537; doi:10.1158/2767-9764.CRC-23-0172)
Supplement: Supplementary Data Figure 5 — Metastatic competency group signature enrichment and patient survival. [file crc-23-0172-s05.pdf]

Supplementary Data Fig. 5: Metastatic competency group signature enrichment and patient survival

**A**

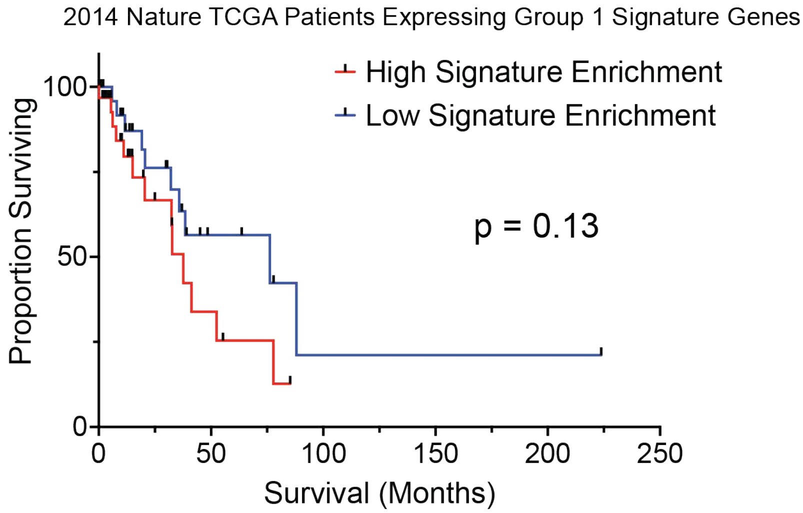


**B**

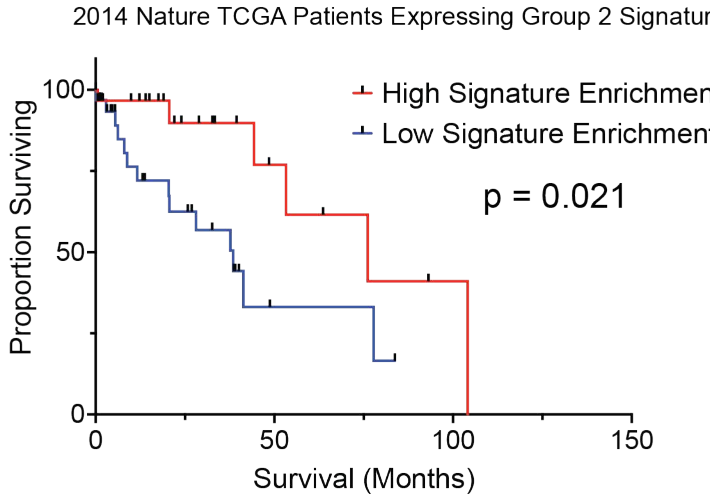


A. Survival curve of patients from Nature 2014 Lung Adenocarcinoma dataset. The red line represents top 15% of patients most enriched for the Group 1 signature genes as measured by single sample gene set enrichment analysis (ssGSEA). The blue line represents those in the bottom 15%. Significance assessed using Kaplan-Meier analysis. The GSVA package in R was used to perform ssGSEA.

B. Analysis as described above for the Group 2 signature genes.
